# Association of regional anesthesia with oncological outcomes in patients receiving surgery for bladder cancer: A meta-analysis of observational studies

**DOI:** 10.3389/fonc.2023.1097637

**Published:** 2023-02-22

**Authors:** Amina M. Illias, Kai-Jie Yu, Shao-Chun Wu, Juan P. Cata, Yung-fong Tsai, Kuo-Chuan Hung

**Affiliations:** ^1^ Department of Anesthesiology, Chang Gung Memorial Hospital, Taoyuan, Taiwan; ^2^ Graduate Institute of Clinical Medical Sciences, College of Medicine, Chang Gung University, Taoyuan, Taiwan; ^3^ Department of Urology, Chang Gung Memorial Hospital, Taoyuan, Taiwan; ^4^ Department of Anesthesiology, Kaohsiung Chang Gung Memorial Hospital and Chang Gung University College of Medicine, Kaohsiung, Taiwan; ^5^ Department of Anesthesiology and Perioperative Medicine, The University of Texas M.D. Anderson Cancer Center, Houston, TX, United States; ^6^ Department of Anesthesiology, Chi Mei Medical Center, Tainan, Taiwan; ^7^ School of Medicine, College of Medicine, National Sun Yat-sen University, Kaohsiung, Taiwan

**Keywords:** regional anesthesia, general anesthesia, bladder cancer, meta-analysis, cancer recurrence, survival rate

## Abstract

**Background:**

This meta-analysis was conducted to compare cancer recurrence and survival rates in patients with bladder cancer receiving surgery under general anesthesia alone (i.e., GA group) or regional anesthesia (RA) with or without GA (i.e., RA ± GA group).

**Methods:**

Literature search on Cochrane library, EMBASE, Google scholar, and Medline databases was performed to identify all relevant studies from inception to April 30, 2022. The primary outcome was cancer recurrence rate, while the secondary outcomes included overall survival rate and cancer-specific survival rate. Subgroup analyses were performed based on study design [(Propensity-score matching (PSM) *vs*. no-PSM)] and type of surgery [transurethral resection of bladder tumor (TURBT) *vs*. radical cystectomy].

**Results:**

Ten retrospective studies with a total of 13,218 patients (RA ± GA group n=4,884, GA group n=8,334) were included. There was no difference between RA ± GA group and GA group in age, the proportion of males, severe comorbidities, the proportion of patients receiving chemotherapy, and the pathological findings (all *p >*0.05). Patients in the RA ± GA group had significantly lower rate of bladder cancer recurrence [odds ratio (OR): 0.74, 95%CI: 0.61 to 0.9, *p*=0.003, I^2 =^ 24%, six studies] compared to those in the GA group. Subgroup analyses based on study design revealed a consistent finding, while the beneficial effect of RA ± GA on reducing cancer recurrence was only significant in patients receiving TURBT (*p*=0.02), but not in those undergoing radical cystectomy (*p*=0.16). There were no significant differences in overall survival rate and cancer-specific survival rate between RA ± GA and GA groups.

**Conclusions:**

For patients receiving surgery for bladder cancer, the application of regional anesthesia with or without general anesthesia is associated with significant decrease in cancer recurrence, especially in patients undergoing TURBT for non-muscle invasive bladder cancer. Because of the limited number of studies included and potential confounding factors, our results should be interpreted carefully.

**Systematic review registration:**

https://www.crd.york.ac.uk/prospero/, identifier CRD42022328134.

## 1 Introduction

Bladder cancer is a common cancer with up to four times higher incidence in men than women (1). Cancer recurrence is still the leading cause of death in those with bladder malignancies (2). When indicated, surgery remains as the preferred therapy in non-metastatic bladder cancers (2). Cancer recurrence and survival rate are affected by many kinds of factors, such as tumor-related factors (3, 4), including circulating tumor DNA, tumor diameter, lymph node metastasis and pathological type. It has been suggested that the choice of anesthesia technique during surgery for cancer resection could affect postoperative oncological outcomes including cancer recurrence (5–7). Many mechanisms have been proposed by which anesthetics could induce immunosuppression and promote cancer metastasis (5, 7).

Regional anesthesia (RA) including nerve and neuraxial blocks with epidural or spinal anesthesia has been associated with improved long-term survival, and fewer recurrences in different cancers (8–10). Numerous amounts of retrospective studies were conducted to compare postoperative outcomes of patients receiving general anesthesia (GA) alone or regional anesthesia with or without general anesthesia (RA ± GA) (5, 8–10). The association between RA and favorable oncological outcomes is yet to be determined. Based on current available data, there are not enough evidence to support the use of RA rather than GA to decrease perioperative immunosuppression, angiogenesis, and cancer metastasis (7).

Regional anesthesia and analgesia are commonly used during bladder cancer surgeries. And it has been reported that in patients undergoing surgery for bladder cancer, RA was associated with lower postoperative cancer recurrence (11) and higher survival rates compared to GA alone (12–14). Nevertheless, when compared to GA, RA during bladder cancer surgery was reported to have worse oncological outcomes (15), increased risk of perioperative complications, higher hospital readmission rates, and longer length of hospital stay (16). Therefore, to incorporate the principle of “do no harm”, it is essential to critically investigate the role of RA in postoperative oncological outcomes after bladder cancer surgery and exclude any possible harm related to intraoperative use of RA.

This meta-analysis aims to evaluate the impact of RA, spinal or epidural, on postoperative oncologic outcomes in bladder cancer patients. We hypothesize that RA could decrease postoperative cancer recurrence in bladder cancer patients.

## 2 Methods

This meta-analysis was conducted in accordance with the Preferred Reporting Items for Systematic reviews and Meta-Analyses (PRISMA) guidelines (17) and registered with the International Prospective Register of Systematic Reviews (CRD42022328134).

### 2.1 Data source and search strategy

Literature search was conducted for all relevant studies on Cochrane library, EMBASE, Google scholar, and Medline databases. The search covered all studies from inception to April 30, 2022 using the following search terms: (“Bladder cancer” or “Bladder Neoplasm*” or “Bladder Tumor*” or “Transurethral Resection of Bladder Tumor*” or “TURBT” or “Cystectomy”) and (“anesthetic technique” or “epidural” or “spinal” or “neuraxial blockade” or “regional anesthesia” or “regional analgesia” or “Extradural Anesthesia” or “Peridural Anesthesia” or “intrathecal”) and (General anesthesia*) and (“Recurrence” or “metastasis” or “Disease-Free Survival” or “Progression-Free Survival” or “Survival” or “Prognosis” or “mortality”). The search strategy for one of these databases are demonstrated in **Supplemental Table 1**.

### 2.2 Eligibility criteria

Studies are considered eligible based on the PICO (population, intervention, comparison, and outcome) format: (P) adults with bladder cancer undergoing a variety of surgeries for bladder cancer treatment, (I) regional anesthesia including spinal and epidural anesthesia with or without administration of general anesthesia (i.e., RA ± GA group), (C) general anesthesia alone with volatile or total intravenous anesthesia and (i.e., GA group) (O) recurrence rate, overall survival rate, and cancer-specific survival rate. References of the retrieved studies were also reviewed for eligibility of being included in the current study.

Only original articles with adult patients (>18years old) who underwent surgery for treatment of bladder cancer were included. Studies presented as letters, abstracts, reviews, and case reports were excluded. Studies that used conscious sedation or peripheral nerve block as the anesthetic technique were excluded. We also excluded studies in which outcomes or anesthesia techniques were not clearly described and studies without a control group. No restriction was placed on gender, study design, study location, language, and sample size during literature search.

### 2.3 Study selection

Study selecting as well as data collecting, and risk assessment were independently performed by two authors. First, the titles and abstracts of all retrieved articles were screened for eligibility of being incorporated into the present study. Next, the full texts of all potentially eligible studies were screened according to the inclusion and exclusion criteria. All disagreements were settled by consulting a third independent reviewer.

### 2.4 Data extraction

The following details were extracted from each report: year of publication, first author, characteristics of participants, sample size, type of surgery, study design [Propensity-score matching (PSM) *vs*. no-PSM], American Society of Anesthesiologists physical status (ASA-PS), proportion of gender, anesthetic techniques, anesthetic agents, recurrence rate, survival-related information, and information regarding histological examination and pathological Tumor-Node-Metastasis (pTNM) Staging if available. Data were extracted and assessed for applicability by two reviewers and discrepancies were settled by a third reviewer. We planned to contact the authors of the included articles if data confirmation was needed in an attempt to access the original data.

### 2.5 Outcomes and subgroup analysis

Patients were divided into two groups based on anesthesia technique, RA ± GA group and GA alone group. The primary outcome was cancer recurrence rate, while the secondary outcomes included overall survival rate and cancer-specific survival rate. Factors such as age, proportion of male gender, severe comorbidities, chemotherapy treatment, and findings on histological examination as well as pTNM Staging were evaluated for significancy between the 2 groups. The definitions of these factors were according to what was originally presented in included studies. Patients with severe comorbidities were defined as those with ASA-PS ≥ 3, Charlson comorbidity score ≥2, age-adjusted comorbidity index≥5, or patients with cerebral vascular disease. Additionally, a subgroup analysis was performed based on study design [PSM *vs*. no-PSM] and type of surgery [transurethral resection of bladder tumor (TURBT) *vs*. radical cystectomy].

### 2.6 Risk of bias assessment and certainty of evidence

The Quality in Prognostic Studies (QUIPS) tool was applied to assess the risk of bias (methodological quality) for the included studies (18–20). Studies were checked by QUIPS tool based on six domains including: study participation, study attrition, prognostic factor measurement, outcome measurement, adjustment for other prognostic factors, and statistical analysis and reporting. Each domain was assigned a low, moderate, or high risk of bias. Studies were defined as having an overall low risk of bias when all the six domains are rated as having low (or low to moderate) risk of bias.

The Grading of Recommendations Assessment, Development and Evaluation (GRADE) (21, 22) was adopted and the certainty of the evidence for our primary and secondary outcomes was assigned to four grades (i.e., high, moderate, low, and very low). Certainty of evidence was evaluated by two independent reviewers based on the probability of study limitations, publication bias, effect consistency, imprecision, and indirectness. All doubts about certainty ratings were settled through discussion.

### 2.7 Data synthesis and analysis

Cochrane Review Manager (RevMan 5.3; Copenhagen: The Nordic Cochrane Centre, The Cochrane Collaboration, 2014) was used for meta-analysis. The pooled results were presented as odds ratios (ORs) and mean difference (MD) with 95% confidence intervals (CIs), respectively. The degree of study heterogeneity was assessed with I^2^ statistics (23). A substantial level of heterogeneity was defined by an I^2^ greater than 50%. The outcomes of the analyses were evaluated using a random effects model on the basis that there is heterogeneity across the studies included in the study. We assessed the potential publication bias of the results by visually inspecting a funnel plot when we encountered ten or more trials reporting the same outcome. Sensitivity analysis was performed with a leave-one-out approach to weigh the potential influence of the data from an individual trial on the overall outcome. The level of significance was set at <0.05 for all outcome analyses.

## 3 Results

### 3.1 Study selection and characteristics

**Figure 1:**
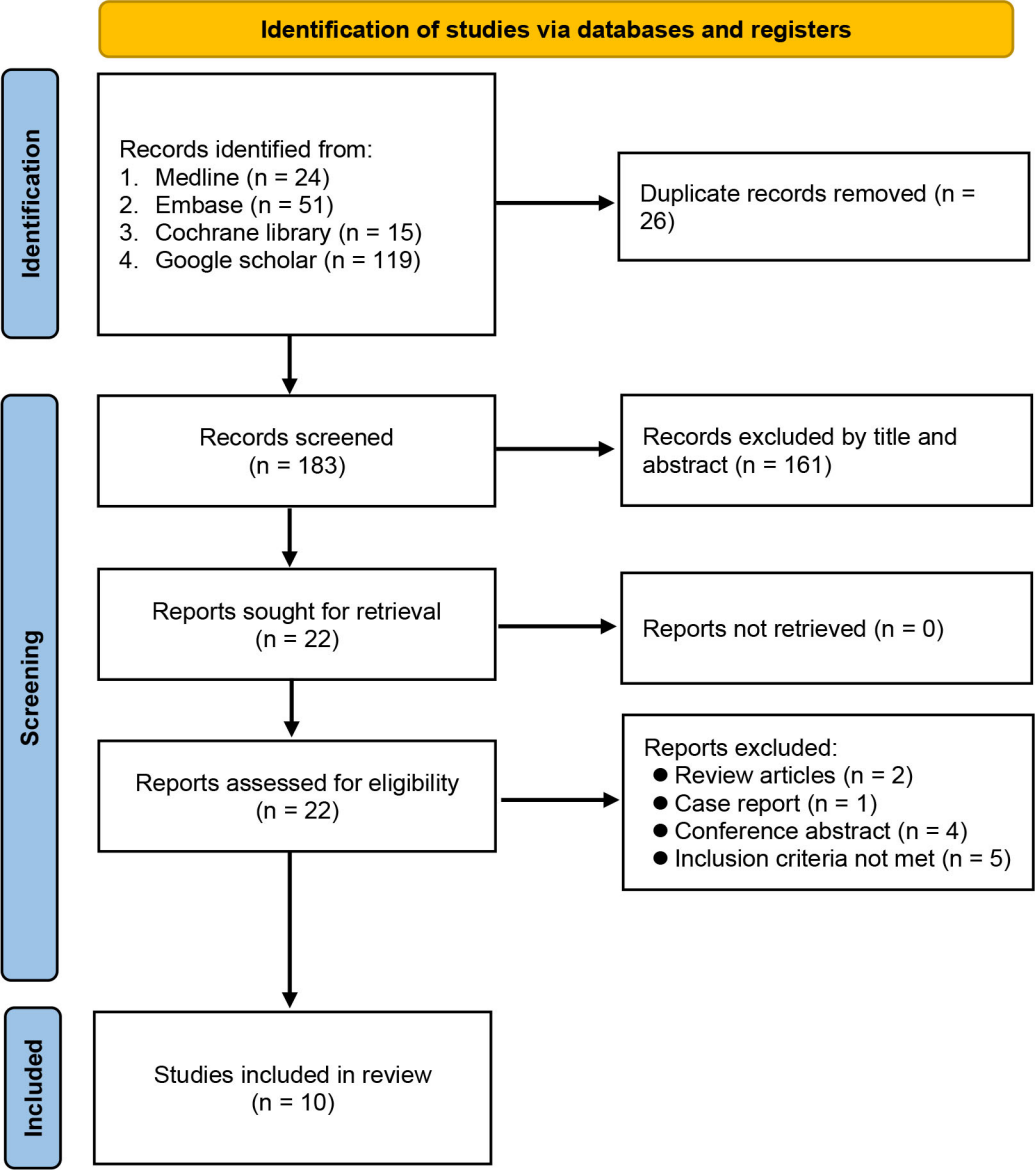
Preferred reporting items for systematic reviews and meta-analyses (PRISMA) flow diagram.

Database searching identified 209 potentially eligible records at initial search from Medline, EMBASE, Cochrane library, and Google scholar. Duplicated records (n=26) and those that did not meet the inclusion criteria by title and abstract (n=161) were excluded. Twenty-two studies with full-texts available were assessed for eligibility. After final evaluation, ten trials were included with a total of 13,218 patients (RA ± GA group n=4,884, GA group n=8,334). The included studies were published from 2016 to 2022 (5, 11–16, 24–26) and according to publication dates, six out of ten studies were published in recent five years (5, 11, 12, 14–16). A flow diagram depicting the process of study selection is demonstrated in **Figure 1**.

All studies were conducted in a retrospective design and study characteristics are listed in **Table 1**. Patients age ranged from 62 to 74 years with wide variation in sample size ranging from 161 to 7,857. The proportion of male gender was between 71% and 84%. Participants received TURBT in six studies (5, 11–14, 24), while radical cystectomy (RC) was performed in four other studies (15, 16, 25, 26). RA was provided in several ways, including spinal anesthesia in four studies (11, 12, 14, 24), epidural anesthesia in one study (16), and in two reports patients received a combination of spinal and epidural anesthesia (5, 13). A combination of GA and RA with either epidural or spinal was reported in three other studies (15, 25, 26). The use of a solution of local anesthetics only was used in four studies (12–14, 24), two studies reported the administration of opioids for RA (15, 26) and four manuscripts did not provide details on the type of anesthetics used for RA (5, 11, 16, 25). As for the GA group, five studies used volatile agents for maintenance (12–14, 24, 26), while other studies did not provide the information (5, 11, 15, 16, 25). In terms of statistical analysis, a PSM was utilized in five of the studies (5, 15, 16, 24, 26). Finally, the included 10 studies were conducted in five countries. Three studies were conducted in Korea (5, 13, 24), two in Canada (11, 25), three in USA (15, 16, 26), one in Japan (12), and one in Turkey (14).

**Table 1:** Characteristics of studies (n = 10).

Study	Year of patient enrollment	Mean Age (years)^a^	Male (%)^a^	Total (n=13,218)	RA (n=4,884)	GA (n=8,334)	RA group	Type of surgery	PSM	Follow-up (years)	Country
Baba 2021	2010-2016	71	78 *vs*. 84	300	147	153	SA	TURBT	No	5	Japan
Chipollini 2018	2008-2012	69 *vs*. 70^b^	73 *vs*. 82	430	215	215	EA+GA	RC	Yes	5	USA
Choi 2017	2000-2007	63 *vs*. 61	83 *vs*. 78	876	718	158	SA	TURBT	Yes	5	Korea
Doiron 2016	2004-2008	NA	79 *vs*. 73	1628	887	741	EA+GA	RC	No	5	Canada
Jang 2016	2001-2008	62 *vs*. 68	78 *vs*. 71	161	137	24	SA/EA	TURBT	No	5	Korea
Koumpan 2018	2011-2013	65 *vs*. 72	81 *vs*. 80	231	135	96	SA	TURBT	No	3-5‡	Canada
Lee 2022	2007-2011	67 *vs*. 67	79 *vs*. 79	1164	582	582	SA/EA	TURBT	Yes	6-10†	Korea
Miller 2020	2002-2014	74 *vs*. 74^b^	80 *vs*. 81	7857	1748	6109	EA	RC	Yes	5	USA
Weingarten 2016	1998-2007	68 *vs*. 68	83 *vs*. 79	390	195	195	SA+GA	RC	Yes	10	USA
Yilmaz 2020	2013-2016	63 *vs*. 61	84 *vs*. 77	181	120	61	SA	TURBT	No	3	Turkey

PSM: Propensity-score matching;

a presented as RA vs. GA;

b presented as median;

RA: regional anesthesia; GA: general anesthesia; RC: Radical cystectomy; TURBT: transurethral resection of bladder tumor; SA: spinal anesthesia; EA: epidural anesthesia; NA: not available;

†Follow-up information was gathered on all patients in September 2017;

‡Follow-up information was gathered on all patients in December 2016.

### 3.2 Risk of bias assessment

**Figure 2:**
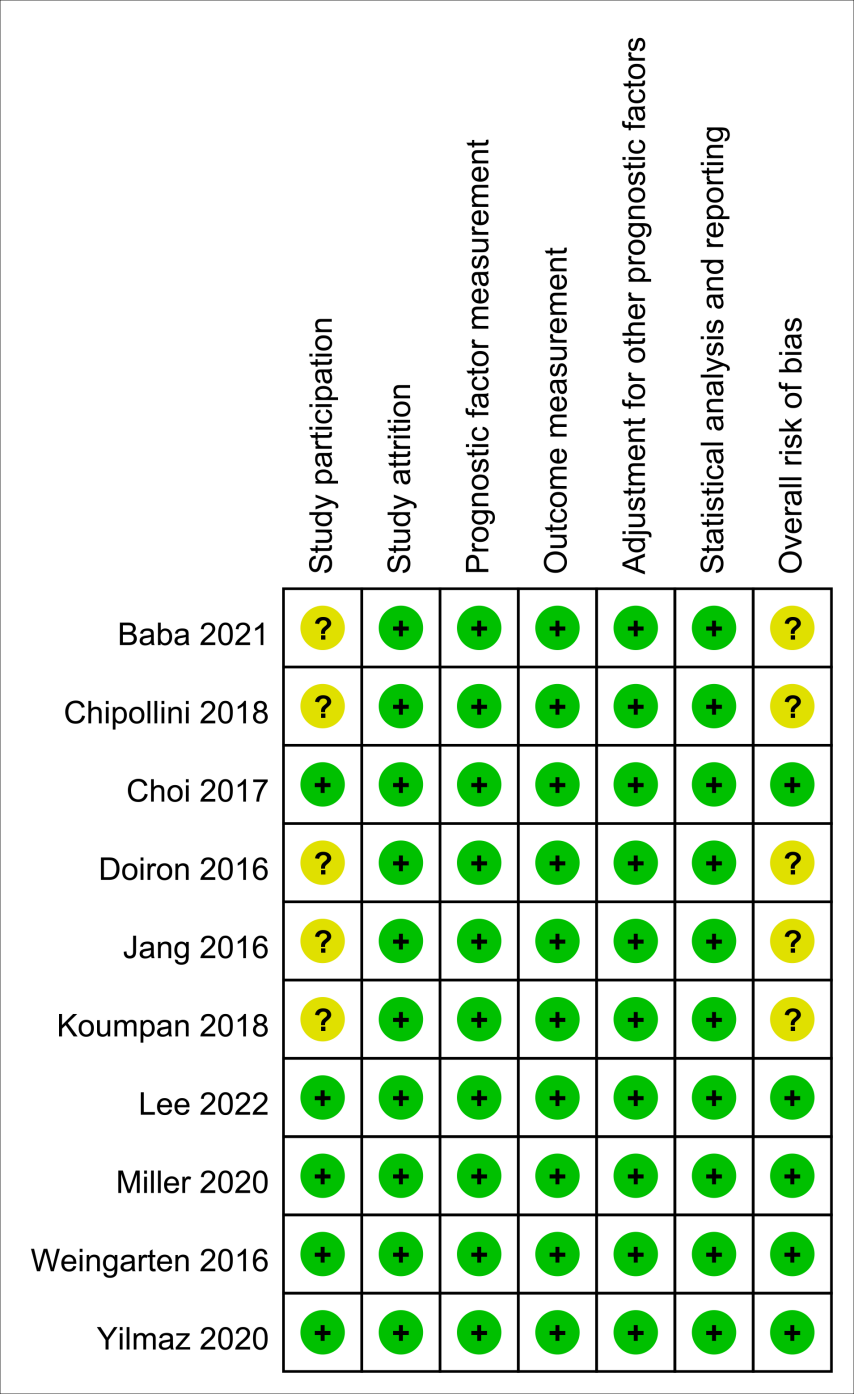
Risk of bias assessment based on Quality in Prognostic Studies (QUIPS) tool. Green: low risk; Yellow: moderate risk.

The risk of bias for all enrolled studies is shown in **Figure 2**. Regarding study participation, moderate risk of bias was considered in five studies due to differences in patient characteristics (e.g., age or male proportion) (11–13, 15, 25). As for the other five studies, risk of bias was considered as low not only for study participation, but also study attrition, prognostic factor measurement, outcome measurement, adjustment for other prognostic factors, and statistical analysis and reporting (5, 14, 16, 24, 26). Therefore, the overall risk of bias was moderate in the five of the studies (11–13, 15, 25), and low in the other five (5, 14, 16, 24, 26).

### 3.3 Results of syntheses

#### 3.3.1 Comparison of baseline characteristics and pathological classification between combined RA ± GA group and GA group

**Figure 3:**
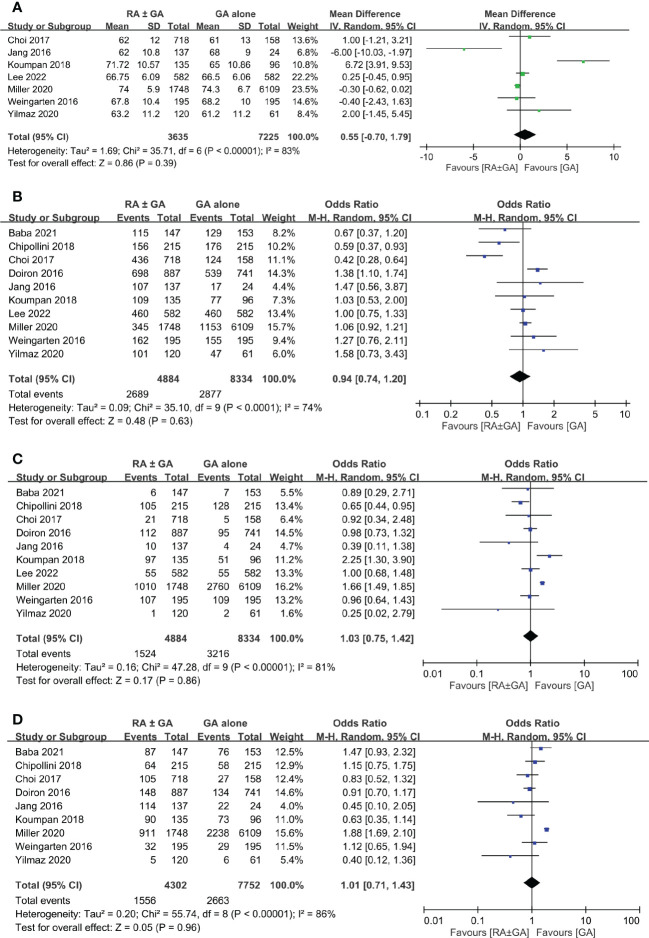
Forest plot showing comparison of **(A)** age; **(B)** male gender; **(C)** severe comorbidities; and **(D)** chemotherapy treatment between RA ± GA group and GA alone group. RA, regional anesthesia; GA, general anesthesia.

**Figure 4:**
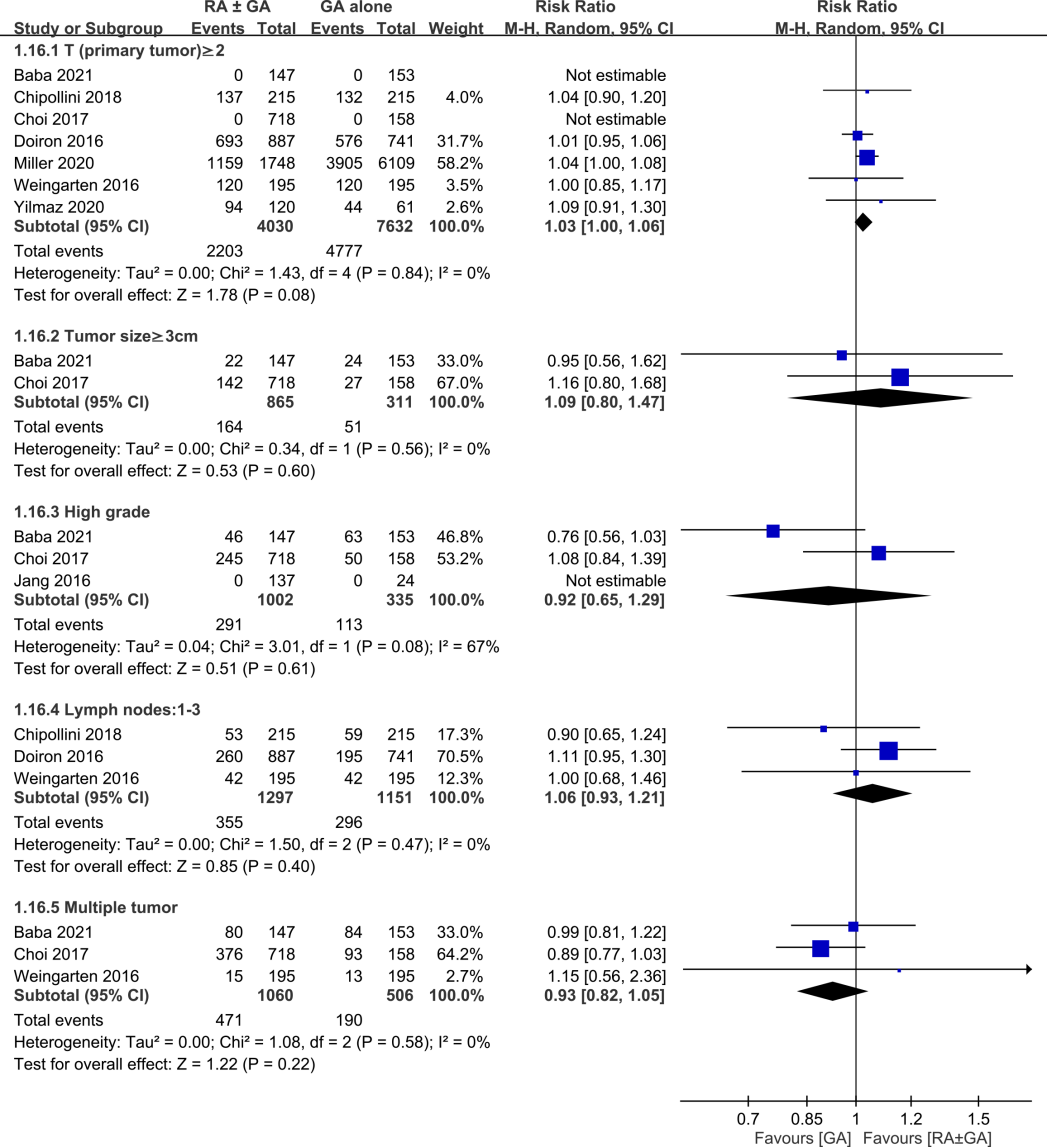
Forest plot showing comparison of pathological findings between RA ± GA group and GA alone group. RA, regional anesthesia; GA, general anesthesia; CI, confidence interval.

There was no difference between RA ± GA group and GA group in age (MD=0.55 years, 95% CI: -0.7 to 1.79, *p*=0.39) (**Figure 3A**), the proportion of male gender (OR=0.94, 95% CI: 0.74 to 1.2, *p*=0.63) (**Figure 3B**), severe comorbidities (OR=1.03, 95% CI: 0.75 to 1.42, *p*=0.86) (**Figure 3C**), and the rate of patients receiving chemotherapy (OR=1.01, 95% CI:0.71 to 1.43, *p*=0.96) (**Figure 3D**). The pathological classification between both groups is demonstrated in **Figure 4**, which showed no difference in tumor-related parameters. Nevertheless, it appeared that the proportion of patients with muscle invasive bladder cancer (i.e., pathological staging T≥2) was higher in the GA group (i.e., 62.6%, 4777/7632) than that in the RA ± GA group (i.e., 54.7%, 2203/4030) without statistical significance.

#### 3.3.2 Primary outcome: Postoperative cancer recurrence rate

**Figure 5:**
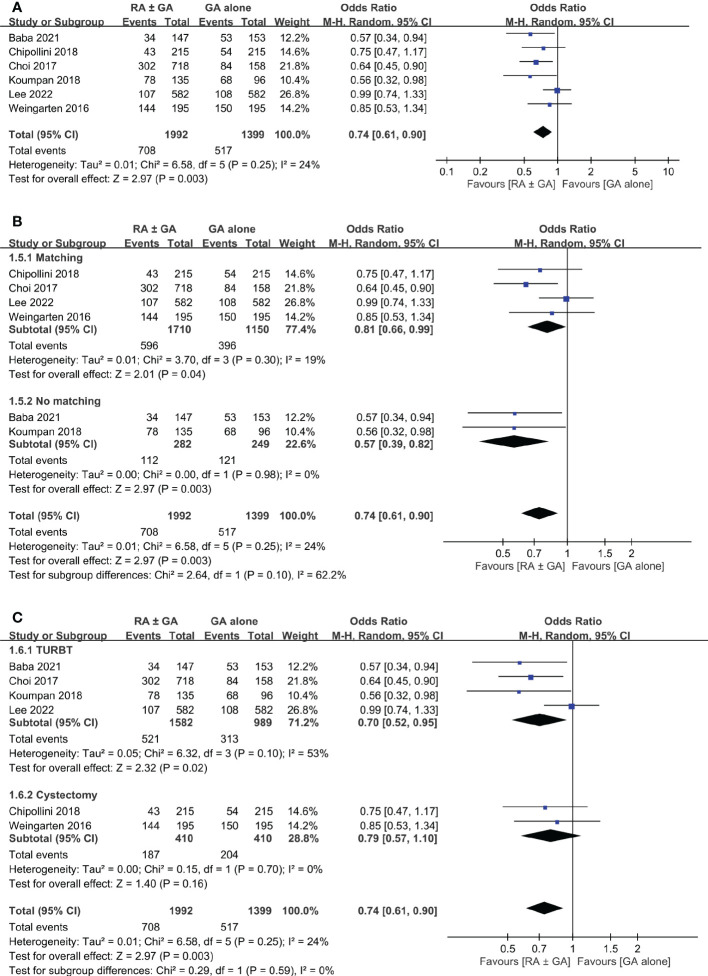
Forest plot showing **(A)** overall recurrence rate in the RA ± GA group compared to the GA alone group; **(B)** subgroup analysis of cancer recurrence rate based on study design; and **(C)** subgroup analysis of cancer recurrence rate based on type of surgery. RA, regional anesthesia; GA, general anesthesia; Matching, used propensity-score matching; No matching, Did not use propensity-score matching; TURBT, transurethral resection of bladder tumor; RC, radical cystectomy.

Six studies involving 3,391 patients provided the information regarding postoperative recurrence rate. Overall, the meta-analysis demonstrated a lower recurrence rate in the RA ± GA group compared to that in the GA group (OR:0.74, 95% CI: 0.61 to 0.9, *p*=0.003, I2 = 24%) (**Figure 5A**).

Subgroup analysis was conducted based on statistical analysis (PSM or not), and type of surgery (TURBT or radical cystectomy). Using PSM had no significant effect on the difference in cancer recurrence rate between the two groups (**Figure 5B**). However, the type of surgical intervention significantly influenced recurrence rate in RA ± GA group (**Figure 5C**). Specifically, the recurrence rate was lower in RA ± GA group undergoing TURBT (OR=0.7, 95% CI: 0.52 to 0.95, *p*=0.02, I^2 =^ 53%), but not in those undergoing radical cystectomy (OR=0.79, 95% CI: 0.57 to 1.1, *p*=0.16, I^2 =^ 0%) (**Figure 5C**).

#### 3.3.3 Secondary outcomes: Overall survival rate and cancer-specific survival rate

**Figure 6:**
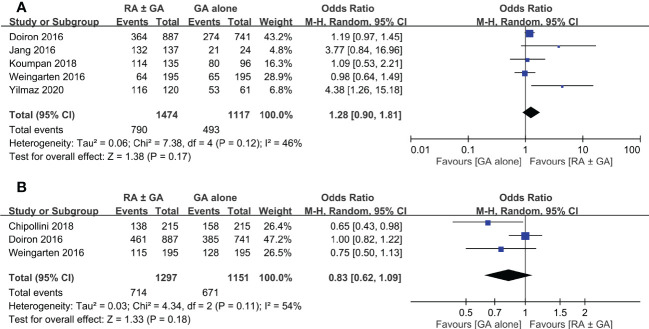
Forest plot showing **(A)** overall survival rate and **(B)** cancer-specific survival rate in the RA ± GA group compared to the GA alone group. RA, regional anesthesia; GA, general anesthesia.

Postoperative overall survival rate was extracted from five studies. There was no significant difference in this outcome between RA ± GA group and GA alone group (OR=1.28, 95% CI: 0.9 to 1.81, *p*=0.17, I^2 =^ 46%) (**Figure 6A**). Three studies comprised details on postoperative cancer-specific survival rate, which again was not impacted by the type of anesthetic techniques (OR=0.83 95% CI: 0.62 to 1.09, *p*=0.18, I^2 =^ 54%) (**Figure 6B**). In addition, one study investigating the impact of epidural anesthesia use on survival outcomes in 7857 patients undergoing radical cystectomy also reported that the use of RA was not associated with decreased overall survival rate (hazard ratio (HR): 0.99, 0.95-1.04, *p*=0.73) or cancer-specific survival rate (HR: 0.96, 0.90-1.02, *p*=0.20) (16).

### 3.4 Sensitivity analysis and evidence of GRADE

Sensitivity analysis demonstrated consistent findings on cancer-related outcomes. The quality of evidence according to the GRADE system is presented in **Supplementary Table 2**. The level of evidence for recurrence rate was graded as low, while the levels of evidence for both overall survival rate and cancer-specific survival rate were graded as very low. The level of evidence was downgraded due to inconsistency or imprecision.

## 4 Discussion

The purpose of this present meta-analysis was to evaluate the impact of neuraxial regional anesthesia on postoperative bladder cancer recurrence, overall survival, and cancer-specific survival. Based on QUIPS tool, risk of bias for included studies were low to moderate. When compared to GA alone, the use of RA ± GA was associated with significantly lower rate of cancer recurrence (*p*=0.003). Based on study design, the RA ± GA group had significantly lower recurrence rate in studies with (*p*=0.003) or without (p=0.04) adopting PSM. However, the beneficial effect of RA was only significant in patients receiving TURBT (*p*=0.02, I^2 =^ 53%), but not in those undergoing radical cystectomy (*p*=0.16).

The choice of anesthetics was reported to influence the course of cancer progression (7, 27–29). Previous studies and meta-analysis suggested that when compared to volatile anesthesia, total intravenous anesthesia with propofol significantly improved recurrence-free survival and overall survival (30–33). The use of GA and perioperative opioids as well as even pain itself could significantly suppress the immune response and influence the proliferation of cancer cells, recurrence, and survival in numerous proposed mechanisms (7, 27–29, 34–38). Nonetheless, it has been theorized that RA could have a positive impact on postoperative oncological outcomes by attenuating the perioperative opioid,GA and surgical stress related immunosuppressive effects (5, 7, 39, 40). Several reviews and meta-analysis were conducted in the past to evaluate the effect of RA on oncological outcomes in patients undergoing cancer resection surgery (35, 39, 41–46). Still, no meta-analysis is available to investigate the impact of RA in bladder cancer solely. A previous meta-analysis had investigated the impact of RA on cancer recurrence where 30% of the included studies were in bladder cancer (41). When several cancers discussed collectively, meta-analysis results neither supported nor strongly advised against the use of RA intraoperatively (35, 41, 44, 46). To the best of our knowledge, this is the first meta-analysis for the influence of RA on postoperative oncological outcomes exclusively in bladder cancer.

Despite our current results, two recent studies suggested that RA for bladder cancer surgery was significantly associated with worse cancer and non-cancer related outcomes (15, 16). For example, one study reported that epidural anesthesia using sufentanil was associated with worse recurrence and disease-free survival (15). While epidural use in another study increased the risk of perioperative complications, hospital readmission and the duration of hospitalization, without improvement in the disease specific survival (16). These findings may not only question the benefit of RA but also alert to the potential harmful effects from using RA for bladder cancer surgery. Compared with patients undergoing TURBT who require minimal postoperative pain control with opioids, it is conceivable that the use of regional anesthesia or analgesia cannot completely obliterate the use of opioids because of the extent of surgery in those receiving radical cystectomy (47). Taking into account the finding of a dose-dependent opioid suppression of the human cellular immune system from a previous study (48), the use of opioids in patients undergoing radical cystectomy may partly explain the lack of significant beneficial impact of RA on their long-term prognosis. Moreover, in contrast to patients subjected to TURBT, those receiving radical cystectomy have a more progressive disease that may overshadow the benefits of RA. In addition, local anesthetics have shown anti-metastatic effects by several mechanisms that involves anti-angiogenic, inhibiting cell migration and invasion (29). Therefore, choosing opioid based epidural (15) or spinal analgesia (26) instead of local anesthetics, may for sure reduce the amount of intravenous opioids but it clearly does not satisfy the concept of using RA to achieve an opioid sparing anesthesia. Adverting to a very important point that we must be mindful of in future research covering the effect of anesthesia technique on postoperative cancer outcomes, which is the type of drug used for RA during cancer resection surgery.

Bladder cancers are staged based on the degree of muscle invasion (2, 49). Most bladder cancers are non-muscle invasive; therefore they can be resected for staging and histological identification by TURBT (49). Studies suggested that TURBT under RA was associated with lower cancer recurrence and better survival rate (11–13, 24). However, RA use for radical cystectomy has no significant impact on cancer recurrence, cancer-specific survival, or overall survival rates (25, 26). Our results were consistent with the findings in previous studies and the association between type of bladder surgery and the beneficial effect from RA in TURBT but not in radical cystectomy could be easily explained by disease severity, stage of cancer and invasiveness at the time of operation (50). Moreover, non-muscle invasive bladder cancer could progress to muscle-invasive cancer (51), requiring longer operation time and much complicated surgical resection with radical cystectomy, which is commonly followed by urinary diversion and neobladder reconstruction. The unfavorable effects of prolonged anesthesia on postoperative non-cancer related outcomes are not discussed in this research. However, for advanced bladder cancer requiring radical cystectomy, it is reasonable to expect higher morbidities and postoperative complications. Future reviews or clinical trials that aim to study the impact of RA or any other anesthesia technique on postoperative bladder cancer recurrence and survival rates, must consider dividing the patients in a more precise way based on cancer invasiveness or type of surgery instead of collectively placing all patients under the same heading, as bladder cancer patients.

This meta-analysis was based on retrospective studies; therefore, it is subject to several limitations. First, although our findings from meta-analysis of observational studies may be useful for identifying associations and generating hypotheses, definite causality cannot be established. Compared with observational studies, randomized controlled trials are more reliable for establishing causality due to a better control for confounding factors to minimize bias. Second, in an effort to limit possible influence by confounding factors that could affect cancer recurrence, we accounted for age, male gender, severe comorbidities, and treatment with chemotherapy. In addition, we minimized the heterogeneity in this meta-analysis by including studies focusing on bladder cancer only. However, other factors could still highly influence our results such as the difference in pathological findings (e.g., pTNM staging), surgical techniques, and the use of different medications and anesthesia techniques during regional or general anesthesia (e.g., total intravenous anesthesia or inhalation anesthesia). As shown in **Figure 4**, despite the lack of statistical significance, the higher proportion of patients with muscle invasive bladder cancer in the GA group than in the RA ± GA group may impair the robustness of our primary outcome. Third, since laparoscopic surgeries are being performed more frequently nowadays, postoperative outcomes after radical cystectomy could also be influenced by adopting laparoscopic or traditional open surgery. Finally, RA provides sufficient anesthesia and pain control during and after the operation and it should be conducted whenever needed. The association between RA and cancer outcomes is still controversial and needs further clinical investigation and carefully planned prospective studies. Based on our search, our results highlighted the crucial need for carefully planned research in the future to increase the level of evidence supporting the beneficial effect of RA in bladder cancer surgery.

In conclusion, this meta-analysis indicates that regional anesthesia with or without general anesthesia is associated with significantly lower recurrence rate in patients with bladder cancer undergoing transurethral resection of bladder tumor. Nevertheless, in light of the limited number of studies included in this review as well as potential confounding factors (e.g., pathological findings), the evidence is not strong enough to support definite conclusions. Until data from prospective randomized controlled trials validate or refute the findings of our research, the use of either GA or RA for bladder cancer is indicated based on patient selection or anesthesiologist’s preference.

## Data availability statement

The original contributions presented in the study are included in the article/**Supplementary Material**. Further inquiries can be directed to the corresponding author.

## Author contributions

Conceptualization: K-CH and S-CW. Methodology: AI, K-JY, Y-fH, and S-CW. Validation: AI, JC, S-CW, and Y-fT. Formal analysis: K-CH, AI, and Y-fT. Investigation: AI, K-JY, Y-fH, and S-CW. Data curation: AI, K-JY, Y-fH, S-CW, and JPC. Writing—original draft preparation: AI, JC, and K-CH. Writing—review and editing: AI and JC. Visualization: AI and K-CH. Supervision: S-CW, JC, and K-CH. All authors contributed to the article and approved the submitted version.

## References

[1] Dobruch J Daneshmand S Fisch M Lotan Y Noon AP Resnick MJ . Gender and bladder cancer: A collaborative review of etiology, biology, and outcomes. Eur Urol. (2016) 69:300–10. doi: 10.1016/j.eururo.2015.08.037 26346676

[2] Lenis AT Lec PM Chamie K Mshs MD . Bladder cancer: A review. JAMA (2020) 324:1980–91. doi: 10.1001/jama.2020.17598 33201207

[3] Chen G Peng J Xiao Q Wu HX Wu X Wang F . Postoperative circulating tumor DNA as markers of recurrence risk in stages II to III colorectal cancer. J Hematol Oncol (2021) 14:80. doi: 10.1186/s13045-021-01089-z 34001194 PMC8130394

[4] Luo X Wu A . Analysis of risk factors for postoperative recurrence of thyroid cancer. J BUON. (2019) 24:813–8. 31128040

[5] Lee SW Tae BS Choi YJ Yoon SM Lee YS Kim JH . A comparison of the anesthetic methods for recurrence rates of bladder cancer after transurethral resection of bladder tumors using national health insurance claims data of south Korea. J Clin Med (2022) 11(4):1143. doi: 10.3390/jcm11041143 35207416 PMC8878593

[6] Kim R Kawai A Wakisaka M Kin T . Current status and prospects of anesthesia and breast cancer: Does anesthetic technique affect recurrence and survival rates in breast cancer surgery? Front Oncol (2022) 12:795864. doi: 10.3389/fonc.2022.795864 35223475 PMC8864113

[7] Kim R . Anesthetic technique and cancer recurrence in oncologic surgery: unraveling the puzzle. Cancer Metastasis Rev (2017) 36:159–77. doi: 10.1007/s10555-016-9647-8 27866303

[8] Biki B Mascha E Moriarty DC Fitzpatrick JM Sessler DI Buggy DJ . Anesthetic technique for radical prostatectomy surgery affects cancer recurrence: a retrospective analysis. Anesthesiology (2008) 109:180–7. doi: 10.1097/ALN.0b013e31817f5b73 18648226

[9] Exadaktylos AK Buggy DJ Moriarty DC Mascha E Sessler DI . Can anesthetic technique for primary breast cancer surgery affect recurrence or metastasis? Anesthesiology (2006) 105:660–4. doi: 10.1097/00000542-200610000-00008 PMC1615712 17006061

[10] Holler JP Ahlbrandt J Burkhardt E Gruss M Rohrig R Knapheide J . Peridural analgesia may affect long-term survival in patients with colorectal cancer after surgery (PACO-RAS-Study): an analysis of a cancer registry. Ann Surg (2013) 258:989–93. doi: 10.1097/SLA.0b013e3182915f61 23629525

[11] Koumpan Y Jaeger M Mizubuti GB Tanzola R Jain K Hosier G . Spinal anesthesia is associated with lower recurrence rates after resection of nonmuscle invasive bladder cancer. J Urol. (2018) 199:940–6. doi: 10.1016/j.juro.2017.11.064 29154849

[12] Baba Y Kikuchi E Shigeta K Ogihara K Matsushima M Nishimoto Y . Effects of transurethral resection under general anesthesia on tumor recurrence in non-muscle invasive bladder cancer. Int J Clin Oncol (2021) 26:2094–103. doi: 10.1007/s10147-021-02000-z 34357470

[13] Jang D Lim CS Shin YS Ko YK Park SI Song SH . A comparison of regional and general anesthesia effects on 5 year survival and cancer recurrence after transurethral resection of the bladder tumor: a retrospective analysis. BMC Anesthesiol. (2016) 16:16. doi: 10.1186/s12871-016-0181-6 26971194 PMC4789273

[14] Yilmaz G . The effect of anesthesia choice on survival in patients undergoing surgery for bladder cancer: A retrospective analysis. Abant Med J (2020) 9(1):1–7. doi: 10.5505/abantmedj.2020.05902

[15] Chipollini J Alford B Boulware DC Forget P Gilbert SM Lockhart JL . Epidural anesthesia and cancer outcomes in bladder cancer patients: is it the technique or the medication? a matched-cohort analysis from a tertiary referral center. BMC Anesthesiol. (2018) 18:157. doi: 10.1186/s12871-018-0622-5 30390636 PMC6215353

[16] Miller BL Abel EJ Allen G Schumacher JR Jarrard D Downs T . Trends in epidural anesthesia use at the time of radical cystectomy and its association with perioperative and survival outcomes: a population-based analysis. Am J Clin Exp Urol. (2020) 8:28–37. 32211451 PMC7076291

[17] Page MJ Moher D Bossuyt PM Boutron I Hoffmann TC Mulrow CD . PRISMA 2020 explanation and elaboration: updated guidance and exemplars for reporting systematic reviews. BMJ (2021) 372:n160. doi: 10.1136/bmj.n160 33781993 PMC8005925

[18] Hayden JA van der Windt DA Cartwright JL Cote P Bombardier C . Assessing bias in studies of prognostic factors. Ann Intern Med (2013) 158:280–6. doi: 10.7326/0003-4819-158-4-201302190-00009 23420236

[19] Lamberink HJ Otte WM Geerts AT Pavlovic M Ramos-Lizana J Marson AG . Individualised prediction model of seizure recurrence and long-term outcomes after withdrawal of antiepileptic drugs in seizure-free patients: a systematic review and individual participant data meta-analysis. Lancet Neurol (2017) 16:523–31. doi: 10.1016/S1474-4422(17)30114-X 28483337

[20] Hayden JA Wilson MN Riley RD Iles R Pincus T Ogilvie R . Individual recovery expectations and prognosis of outcomes in non-specific low back pain: prognostic factor review. Cochrane Database Syst Rev (2019) 2019(11):CD011284. doi: 10.1002/14651858.CD011284.pub2 31765487 PMC6877336

[21] Guyatt GH Oxman AD Kunz R Vist GE Falck-Ytter Y Schunemann HJ . What is “quality of evidence” and why is it important to clinicians? BMJ (2008) 336:995–8. doi: 10.1136/bmj.39490.551019.BE PMC2364804 18456631

[22] Guyatt GH Oxman AD Vist GE Kunz R Falck-Ytter Y Alonso-Coello P . GRADE: an emerging consensus on rating quality of evidence and strength of recommendations. BMJ (2008) 336:924–6. doi: 10.1136/bmj.39489.470347.AD PMC2335261 18436948

[23] Higgins JP Thompson SG Deeks JJ Altman DG . Measuring inconsistency in meta-analyses. BMJ (2003) 327:557–60. doi: 10.1136/bmj.327.7414.557 PMC192859 12958120

[24] Choi WJ Baek S Joo EY Yoon SH Kim E Hong B . Comparison of the effect of spinal anesthesia and general anesthesia on 5-year tumor recurrence rates after transurethral resection of bladder tumors. Oncotarget (2017) 8:87667–74. doi: 10.18632/oncotarget.21034 PMC5675662 29152110

[25] Doiron RC Jaeger M Booth CM Wei X Siemens DR . Is there a measurable association of epidural use at cystectomy and postoperative outcomes? a population-based study. Can Urological Assoc J (2016) 10:321. doi: 10.5489/cuaj.3856 PMC5085911 27800053

[26] Weingarten TN Taccolini AM Ahle ST Dietz KR Dowd SS Frank I . Perioperative management and oncological outcomes following radical cystectomy for bladder cancer: a matched retrospective cohort study. Can J Anaesth. (2016) 63:584–95. doi: 10.1007/s12630-016-0599-9 26850064

[27] Buddeberg BS Seeberger MD . Anesthesia and oncology: Friend or foe? Front Oncol (2022) 12:802210. doi: 10.3389/fonc.2022.802210 35359377 PMC8963958

[28] Kim R . Effects of surgery and anesthetic choice on immunosuppression and cancer recurrence. J Transl Med (2018) 16:8. doi: 10.1186/s12967-018-1389-7 29347949 PMC5774104

[29] Cata JP Guerra C Soto G Ramirez MF . Anesthesia options and the recurrence of cancer: What we know so far? Local Reg Anesth (2020) 13:57–72. doi: 10.2147/LRA.S240567 32765061 PMC7369361

[30] Pfail JL Katims AB Gul Z Rosenzweig SJ Razdan S Nathaniel S . Can anesthetics affect bladder cancer recurrence? total intravenous versus volatile anesthesia in patients undergoing robot-assisted radical cystectomy: A single institution retrospective analysis. Urol Oncol (2021) 39:233.e1–e8. doi: 10.1016/j.urolonc.2020.08.024 32951989

[31] Wigmore TJ Mohammed K Jhanji S . Long-term survival for patients undergoing volatile versus IV anesthesia for cancer surgery: A retrospective analysis. Anesthesiology (2016) 124:69–79. doi: 10.1097/ALN.0000000000000936 26556730

[32] Jin Z Li R Liu J Lin J . Long-term prognosis after cancer surgery with inhalational anesthesia and total intravenous anesthesia: a systematic review and meta-analysis. Int J Physiol Pathophysiol Pharmacol (2019) 11:83–94. 31333811 PMC6628011

[33] Yap A Lopez-Olivo MA Dubowitz J Hiller J Riedel B . Global onco-anesthesia research collaboration g. anesthetic technique and cancer outcomes: a meta-analysis of total intravenous versus volatile anesthesia. Can J Anaesth. (2019) 66:546–61. doi: 10.1007/s12630-019-01330-x 30834506

[34] Novy DM Nelson DV Koyyalagunta D Cata JP Gupta P Gupta K . Pain, opioid therapy, and survival: a needed discussion. Pain (2020) 161:496–501. doi: 10.1097/j.pain.0000000000001736 31693537 PMC7017938

[35] Lusty AJ Hosier GW Koti M Chenard S Mizubuti GB Jaeger M . Anesthetic technique and oncological outcomes in urology: A clinical practice review. Urol Oncol (2019) 37:845–52. doi: 10.1016/j.urolonc.2019.08.004 31526652

[36] Missair A Cata JP Votta-Velis G Johnson M Borgeat A Tiouririne M . Impact of perioperative pain management on cancer recurrence: an ASRA/ESRA special article. Reg Anesth Pain Med (2019) 44:13–28. doi: 10.1136/rapm-2018-000001 30640648

[37] Dubowitz J Hiller J Riedel B . Anesthetic technique and cancer surgery outcomes. Curr Opin Anaesthesiol. (2021) 34:317–25. doi: 10.1097/ACO.0000000000001002 33935180

[38] Dubowitz JA Sloan EK Riedel BJ . Implicating anaesthesia and the perioperative period in cancer recurrence and metastasis. Clin Exp Metastasis. (2018) 35:347–58. doi: 10.1007/s10585-017-9862-x 28894976

[39] Lee BM Singh Ghotra V Karam JA Hernandez M Pratt G Cata JP . Regional anesthesia/analgesia and the risk of cancer recurrence and mortality after prostatectomy: a meta-analysis. Pain Manage (2015) 5:387–95. doi: 10.2217/pmt.15.30 PMC5561907 26250850

[40] Cata JP . Outcomes of regional anesthesia in cancer patients. Curr Opin Anaesthesiol. (2018) 31:593–600. doi: 10.1097/ACO.0000000000000636 30020153

[41] Ang E Ng KT Lee ZX Ti LK Chaw SH Wang CY . Effect of regional anaesthesia only versus general anaesthesia on cancer recurrence rate: A systematic review and meta-analysis with trial sequential analysis. J Clin Anesth (2020) 67:110023. doi: 10.1016/j.jclinane.2020.110023 32805685

[42] Perez-Gonzalez O Cuellar-Guzman LF Navarrete-Pacheco M Ortiz-Martinez JJ Williams WH Cata JP . Impact of regional anesthesia on gastroesophageal cancer surgery outcomes: A systematic review of the literature. Anesth Analg. (2018) 127:753–8. doi: 10.1213/ANE.0000000000003602 29958224

[43] Perez-Gonzalez O Cuellar-Guzman LF Soliz J Cata JP . Impact of regional anesthesia on recurrence, metastasis, and immune response in breast cancer surgery: A systematic review of the literature. Reg Anesth Pain Med (2017) 42:751–6. doi: 10.1097/AAP.0000000000000662 28953508

[44] Grandhi RK Lee S Abd-Elsayed A . The relationship between regional anesthesia and cancer: A metaanalysis. Ochsner J (2017) 17:345–61. PMC5718448 29230120

[45] Sun Y Li T Gan TJ . The effects of perioperative regional anesthesia and analgesia on cancer recurrence and survival after oncology surgery: A systematic review and meta-analysis. Reg Anesth Pain Med (2015) 40:589–98. doi: 10.1097/AAP.0000000000000273 26263074

[46] Pei L Tan G Wang L Guo W Xiao B Gao X . Comparison of combined general-epidural anesthesia with general anesthesia effects on survival and cancer recurrence: a meta-analysis of retrospective and prospective studies. PloS One (2014) 9:e114667. doi: 10.1371/journal.pone.0114667 25548913 PMC4280190

[47] Karadeniz MS Mammadov O Çiftci H Usta SA Pembeci K . Comparing the effects of combined General/Epidural anaesthesia and general anaesthesia on serum cytokine levels in radical cystectomy. Turkish J anaesthesiol reanimation. (2017) 45:203–9. doi: 10.5152/TJAR.2017.13285 PMC5579213 28868167

[48] Yeager MP Colacchio TA Yu CT Hildebrandt L Howell AL Weiss J . Morphine inhibits spontaneous and cytokine-enhanced natural killer cell cytotoxicity in volunteers. Anesthesiology (1995) 83:500–8. doi: 10.1097/00000542-199509000-00008 7661350

[49] DeGeorge KC Holt HR Hodges SC . Bladder cancer: Diagnosis and treatment. Am Fam Physician. (2017) 96:507–14. 29094888

[50] Sun M Abdollah F Bianchi M Trinh QD Shariat SF Jeldres C . Conditional survival of patients with urothelial carcinoma of the urinary bladder treated with radical cystectomy. Eur J Cancer. (2012) 48:1503–11. doi: 10.1016/j.ejca.2011.11.024 22196034

[51] Sylvester RJ van der Meijden AP Oosterlinck W Witjes JA Bouffioux C Denis L . Predicting recurrence and progression in individual patients with stage Ta T1 bladder cancer using EORTC risk tables: a combined analysis of 2596 patients from seven EORTC trials. Eur Urol. (2006) 49:466–5. doi: 10.1016/j.eururo.2005.12.031 16442208

