# Supplementary material for: Association of regional anesthesia with oncological outcomes in patients receiving surgery for bladder cancer: A meta-analysis of observational studies
Source: Front Oncol. 2023 Feb 22;13:1097637. doi: 10.3389/fonc.2023.1097637 (PMC9992181; doi:10.3389/fonc.2023.1097637)
Supplement: Supplementary file 1 [file DataSheet_1.docx]

**Supplementary Table 1.** Search strategies for Medline

| 1 | ("Bladder cancer" or "Bladder Neoplasm*" or "Bladder Tumor*" or "Transurethral Resection of Bladder Tumor*" or "TURBT" or "Cystectomy").mp. |
| --- | --- |
| 2 | exp "Urinary Bladder Neoplasms"/ |
| 3 | ("anaesthetic technique" or "epidural" or "spinal" or "neuraxial blockade" or "regional anesthesia" or "regional analgesia" or "Extradural Anesthesia" or "Peridural Anesthesia" or "intrathecal").mp. |
| 4 | exp "Analgesia, Epidural"/ or exp "Anesthesia, Epidural"/ or exp "Anesthesia, Spinal"/ or exp "Anesthesia, Conduction"/ |
| 5 | General anesthesia*.mp. |
| 6 | exp "Anesthesia, General"/ |
| 7 | ("Recurrence" or "metastasis" or "Disease-Free Survival" or "Progression-Free Survival" or "Survival" or "Prognosis" or "mortality").mp. |
| 8 | exp "Neoplasm Metastasis"/ or exp "Disease-Free Survival"/ or exp "Progression-Free Survival"/ or exp "Mortality"/ or exp "Neoplasm Recurrence, Local"/ |
| 9 | (1 or 2) and (3 or 4) and (5 or 6) and (7 or 8) |

**Supplementary table 2:** The quality of evidence according to the GRADE system

|  | | | | | | |
| --- | --- | --- | --- | --- | --- | --- |
| Outcomes | **Effect (Risk)** | | Relative effect (95% CI) | № of participants  (studies) | Certainty of the evidence (GRADE) | Comments |
|  | **Intervention group** | **Control group** |  |  |  |  |
| Recurrence rate | 708/1992 | 517/1399 | **RR 0.74** (0.61 to 0.9) | 3391 (6 RCTs) | ⨁⨁◯◯ Low | - |
| Overall survival rate | 790/1474 | 493/1117 | **MD 1.28** (0.9 to 1.81) | 2591 (5 RCTs) | ⨁◯◯◯ Very Low | a |
| Cancer-specific survival rate | 714/1297 | 671/1151 | **RR 0.83** (0.62 to 1.09) | 2448 (3 RCTs) | ⨁◯◯◯ Very Low | a,b |

^a^wide 95% CI

^b^The I square is more than 50%.

**GRADE Working Group grades of evidence:
High certainty**: We are very confident that the true effect lies close to that of the estimate of the effect
**Moderate certainty**: We are moderately confident in the effect estimate: The true effect is likely to be close to the estimate of the effect, but there is a possibility that it is substantially different
**Low certainty**: Our confidence in the effect estimate is limited: The true effect may be substantially different from the estimate of the effect
**Very low certainty**: We have very little confidence in the effect estimate: The true effect is likely to be substantially different from the estimate of effect
